# Assessing genomic diversity and signatures of selection in Jiaxian Red cattle using whole-genome sequencing data

**DOI:** 10.1186/s12864-020-07340-0

**Published:** 2021-01-09

**Authors:** Xiaoting Xia, Shunjin Zhang, Huaju Zhang, Zijing Zhang, Ningbo Chen, Zhigang Li, Hongxia Sun, Xian Liu, Shijie Lyu, Xianwei Wang, Zhiming Li, Peng Yang, Jiawei Xu, Xiaoting Ding, Qiaoting Shi, Eryao Wang, Baorui Ru, Zejun Xu, Chuzhao Lei, Hong Chen, Yongzhen Huang

**Affiliations:** 1 Key Laboratory of Animal Genetics, Breeding and Reproduction of Shaanxi Province, College of Animal Science and Technology, Northwest A&F University, No. 22 Xinong Road, Yangling, 712100 Shaanxi China; 2 Pingdingshan animal husbandry technology promotion station, Pingdingshan, 467000 Henan China; 3 Institute of Animal Husbandry and Veterinary Science, Henan Academy of Agricultural Sciences, Zhengzhou, 450002 Henan China; 4 Henan Provincial Animal Husbandry General Station, Zhengzhou, 450008 Henan China

**Keywords:** Chinese cattle, Genetic diversity, Population structure, Genetic signatures, *Bos taurus*, *Bos indicus*

## Abstract

**Background:**

Native cattle breeds are an important source of genetic variation because they might carry alleles that enable them to adapt to local environment and tough feeding conditions. Jiaxian Red, a Chinese native cattle breed, is reported to have originated from crossbreeding between taurine and indicine cattle; their history as a draft and meat animal dates back at least 30 years. Using whole-genome sequencing (WGS) data of 30 animals from the core breeding farm, we investigated the genetic diversity, population structure and genomic regions under selection of Jiaxian Red cattle. Furthermore, we used 131 published genomes of world-wide cattle to characterize the genomic variation of Jiaxian Red cattle.

**Results:**

The population structure analysis revealed that Jiaxian Red cattle harboured the ancestry with East Asian taurine (0.493), Chinese indicine (0.379), European taurine (0.095) and Indian indicine (0.033). Three methods (nucleotide diversity, linkage disequilibrium decay and runs of homozygosity) implied the relatively high genomic diversity in Jiaxian Red cattle. We used θπ, CLR, *F*_ST_ and XP-EHH methods to look for the candidate signatures of positive selection in Jiaxian Red cattle. A total number of 171 (θπ and CLR) and 17 (*F*_ST_ and XP-EHH) shared genes were identified using different detection strategies. Functional annotation analysis revealed that these genes are potentially responsible for growth and feed efficiency (*CCSER1*), meat quality traits (*ROCK2*, *PPP1R12A, CYB5R4*, *EYA3*, *PHACTR1*), fertility (*RFX4*, *SRD5A2*) and immune system response (*SLAMF1*, *CD84* and *SLAMF6*).

**Conclusion:**

We provide a comprehensive overview of sequence variations in Jiaxian Red cattle genomes. Selection signatures were detected in genomic regions that are possibly related to economically important traits in Jiaxian Red cattle. We observed a high level of genomic diversity and low inbreeding in Jiaxian Red cattle. These results provide a basis for further resource protection and breeding improvement of this breed.

**Supplementary Information:**

The online version contains supplementary material available at 10.1186/s12864-020-07340-0.

## Background

Domesticated cattle can be categorized into two subspecies: humpless taurine (*Bos taurus*) and humped indicine (*Bos indicus*) with some subsequent hybridization events resulting in the descendant hybrid breeds being adapted to various environments [1]. Whole-genome sequencing (WGS) has been used to detect population structure and identify polymorphisms that might affect the economic traits of livestock animals. Recently, the WGS analysis of domestic cattle revealed that the worldwide cattle could be divided into five continental groups: European taurine, Eurasian taurine, East Asian taurine, Chinese indicine and Indian indicine [2]. Based on WGS many studies initially focused on the genetic architecture and economic traits under positive selection of European commercial breeds [3, 4], and then gradually focused on the adaptable indigenous breeds, such as African cattle [5, 6]. They identified genomic variations characteristics (new genes or genetic pathways) of native cattle adapting to local environments, such as climate challenges and disease resistance [5, 6]. These evidences can provide options for designing genetic breeding strategies to improve the adaptability and productivity of cattle. However, there are a limited number of studies on the genomic variation of native cattle breeds in China, and even fewer reports have been reported from Jiaxian Red cattle [2, 7, 8].

Jiaxian Red is an indigenous cattle breed in China, and it has been intensively bred for beef during over the past 30 years, leading to genetic improvement in production traits [9]. In the early production practice, the local people mainly used the draught as the selection standard, which led to the large body size of Jiaxian Red cattle. For now, Jiaxian Red cattle have been evolved into a beef cattle breed with excellent meat quality, tough feeding resistance and high fertility [10]. Previous studies have used various methods to study the genomic diversity and population structure of the Jiaxian Red cattle breed, e.g., mitochondrial, Y chromosome or autosomal data [2, 11, 12]. These studies indicated that Jiaxian Red originated from the crossbreeding between taurine and indicine cattle. Studies attempting to explain the growth and meat quality of Jiaxian Red cattle have focused on single gene SNPs (single nucleotide polymorphisms), such as *MYLK4* [13], *TRPV1* and *TRPA1* [14], and *CRTC3* [15]. However, there has been no previous studies using WGS data to identify genes under the selection pressure of this breed.

To expand our knowledge of genomic variations and selective sweeps that may potentially have arisen as a result of recent selection of Jiaxian Red, we performed whole-genome sequencing of 30 Jiaxian Red cattle and identified SNPs based on the *Bos taurus* reference genome assembly (ARS-UCD1.2). SNPs of Jiaxian Red were compared with those of commercial and native breeds previously collected from around the world.

## Results

### Sequencing, assembly, and identification of single nucleotide polymorphisms

Individual genomes of 30 Jiaxian Red cattle were generated to ~ 10.6 × coverage each and were jointly genotyped with publicly available genomes of five “core” cattle populations [2] and Qinchuan breed (Chinese native beef cattle) (Tables S1 and S2). Five “core” cattle populations comprise European taurine (Hereford and Angus), Eurasian taurine (Gelbvieh, Limousin, Simmental and Jersey), East Asian taurine (Hanwoo, Mishima and Tibetan), Chinese indicine (Wannan, Guangfeng, Ji’an, Leiqiong) and Indian indicine (Tharparkar, Nelore, Sahiwal, Hariana and Gir). In total, ~ 5.0 billion reads of sequences were generated. Using BWA-MEM (0.7.13-r1126), reads were aligned to the *Bos taurus* reference genome sequence (ARS-UCD1.2) with an average of 11.5 × coverage. We annotated 24,800,431 biallelic SNPs that were discovered in 30 Jiaxian Red cattle. Functional annotation of the polymorphic sites revealed that the vast majority of SNPs were present either intergenic regions (59.2%) or intronic regions (37.8%). Exons contained 0.8% of the total SNPs with 70,165 non-synonymous SNPs and 112,847 synonymous SNPs (Table S3).

The total number of SNPs detected within the breeds was showed in Table S3. The Chinese indicine (29,715,667; *Bos indicus*) displayed the highest number of SNP, followed by crossbred Jiaxian Red (24,800,431; *Bos taurus* × *Bos indicus*), Indian indicine (21,149,877; *Bos indicus*) and Qinchuan cattle (20,233,594; *Bos taurus* × *Bos indicus*). As expected, the SNPs of taurine cattle were significantly lower than that of hybrid and zebu breeds. This distribution pattern of SNPs is consistent with that reported in previous study [2].

### Population structure and relationships

**Fig. 1:**
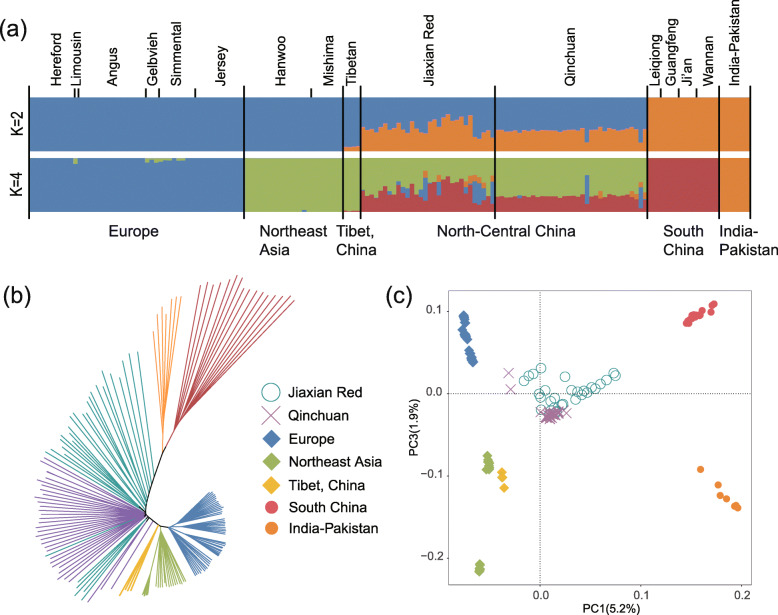
Population structure and relationships of Jiaxian Red in comparison to several possible ancestral breeds. **a** Model-based clustering of cattle breeds using ADMIXTURE with K = 2 and K = 4. Breeds are colored by geographic regions and labelled with breed name. **b** Neighbor-joining tree of the relationships between the ten cattle breeds (161 animals). **c** Principal component analysis of 10 cattle breeds

To explore relatedness among Jiaxian Red cattle and other cattle breeds distributed worldwide, we conducted ADMIXTURE, neighbor-joining (NJ) and principle component analysis (PCA) using genomic SNPs (Fig. 1). The analyses revealed clear geographic patterns among cattle populations as previous suggested [2]. In the ADMIXTURE analysis, when K=2, the cattle breeds were genetically divided into *Bos taurus* and *Bos indicus* ancestry; when K=4, the Jiaxian Red cattle showed clear evidence of genetic heterogeneity with shared genome ancestry with East Asian taurine (0.493), Chinese indicine (0.379), European taurine (0.095) and Indian indicine (0.033) genetic background (Fig. 1a, K=4). The genetic influence of *Bos indicus* was greater on Jiaxian Red than that on Qinchuan cattle, with an average genetic proportion of 0.412 and 0.292, respectively. The NJ tree and PCA analysis provided similar results, with all the “core” cattle populations forming their own separate clusters, and Jiaxian Red as well as Qinchuan cattle are found at an intermediate position between *Bos taurus* and *Bos indicus* (Fig. 1b and c).

### Patterns of genomic variation

**Fig. 2:**
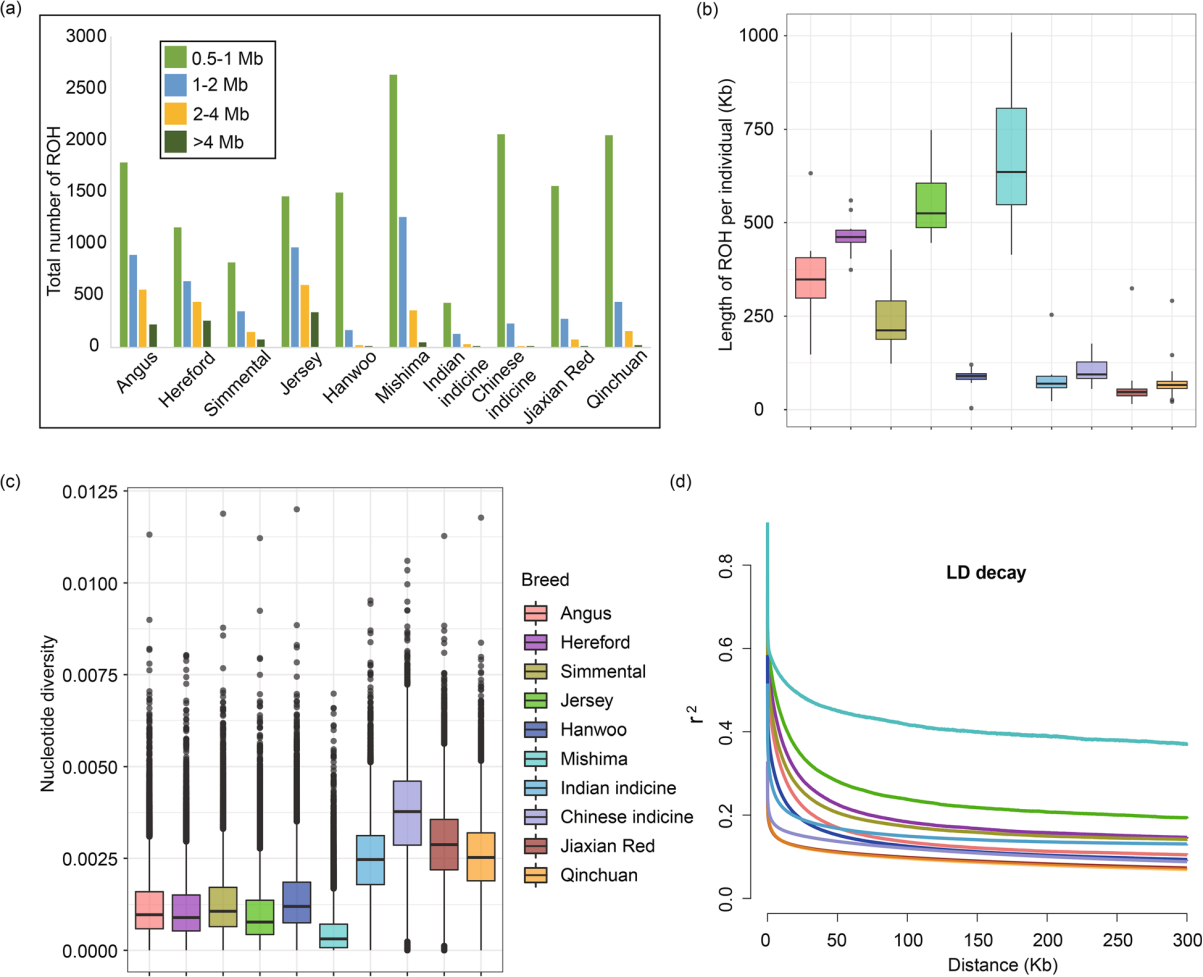
Summary statistics for genomic variation. **a** The distribution of total number of ROH across chromosomes. **b** The distribution of lengths ROH in each breed. **c** Genome-wide distribution of nucleotide diversity of each breed in 50 kb windows with 50 kb steps. The horizontal line inside the box indicates the median of this distribution; box limits indicate the first and the thirds quartiles, points shows outliers. Data points outside the whiskers can be considered as outliers. **d** Genome-wide average LD decay estimated from each breed

Runs of homozygosity (ROH) are continuous homozygous regions in the DNA sequence of diploid organisms [16]. To evaluate the ROH pattern of Jiaxian Red and other cattle breeds, we divided the length of ROH into four size classes: 0.5–1 Mb, 1–2 Mb, 2-4 Mb, > 4 Mb (Fig. 2a). The presence of long ROH is the result of consanguineous mating, whereas shorter ROH reflect distant ancestral influences [17]. The vast majority of ROH that identified in all breeds are between 0.5–1 Mb in length, but apparently European commercial breeds have more medium (2–4 Mb) and long ROH (> 4 Mb). The total lengths of ROHs in Mishima and European taurine breeds (Jersey, Hereford, Angus and Simmental) are longer than those of Jiaxian Red because European commercial breeds have been artificially selected for a longer period of time (Fig. 2b). In Fig. 2c, our results showed that nucleotide diversity was the highest in Chinese indicine cattle, followed by Jiaxian Red, Qinchuan and Indian indicine cattle. The lowest nucleotide diversity was found for European and East Asian taurine cattle. In contrast, we observed the lowest average genome-wide linkage disequilibrium (LD) in Jiaxian Red and Qinchuan cattle and the highest value of LD in Mishima, followed by European taurine (Jersey, Hereford, Simmental and Angus), Hanwoo and zebu cattle (Indian and Chinese indicine) (Fig. 2d).

### Functional enrichment analysis of the specific SNPs in Jiaxian red

When compared to five “core” cattle breeds, a total of 1,817,304 SNPs were found specific to Jiaxian Red, which is lower than the zebu cattle groups but higher than the taurine cattle groups (Figure S1). When comparing the two Chinese cattle breeds, Jiaxian Red and Qinchuan cattle shared a relatively high number of SNPs (*n*=17,828,430), accounting for ~ 72% and ~ 88% of the total SNPs of Jiaxian Red and Qinchuan respectively, indicating that the genetic relationship between the two cattle breeds is very close.

In order to find out the genetic differences between Jiaxian Red and Qinchuan cattle, ANNOVAR software was used to identify the nonsynonymous SNPs (nsSNPs) of breeds. We obtained 20,445 and 7266 specific nsSNPs in Jiaxian Red and Qinchuan breeds, respectively. Following the methods by Kawahara-Miki et al. (2011) [18] and Weldenegodguad et al. (2019) [19], we selected genes containing > 5 nsSNPs for each breed. Finally, a total of 617 and 88 genes were identified in Jiaxian Red and Qinchuan. From DAVID gene ontology, 21 significant (*P*< 0.05) GO BP terms were enriched in Jiaxian Red (Figure S2 and Table S4). The most significant term was associated with immune function (Antigen processing and presentation of peptide antigen via MHC class I, GO:0002474), including one gene *BOLA*. Three terms were associated with sensory perception functions, such as “Photoreceptor cell maintenance, GO:0045494”, “Sensory perception of light stimulus, GO:0050953” and “Sensory perception of sound, GO:0007605”. Among the other genes were those related to molecular functions that might contribute to the specific characteristics of the Jiaxian Red breed. In Qinchuan cattle, four significant (*P*< 0.05) GO BP terms were enriched, including biological process “Cilium assembly, GO:0042384”, “Epithelial cilium movement, GO:0003351”, “Regulation of centrosome duplication, GO:0010824” and “Positive regulation of intracellular protein transport, GO:0090316” (Figure S3 and Table S5).

### Genome-wide selective sweep test

We applied the nucleotide diversity analysis (θπ) and the composite likelihood ratio (CLR) methods to detect genomic regions related to selection in Jiaxian Red breed. Two methods showed outlier signals (top 1%) in overlapping regions and were therefore considered as candidate selective regions. A total of 1199 (θπ) and 351 (CLR) genes with selection signatures in Jiaxian Red cattle were identified, 171 of which were overlapped (Tables S6 and S7). We performed functional enrichment analysis using KEGG pathways and Gene Ontology (GO) for overlapped genes. The only significant KEGG pathway in Jiaxian Red was “Regulation of actin cytoskeleton” (corrected *P-*value *<* 0.05, Table S8) involving 6 genes (*ITGA1*, *ENAH*, *MYLK3*, *ROCK2*, *PFN4*, *PPP1R12A*), which is related to meat tenderness [20, 21], feed efficiency and compensatory gain in cattle [22]. Over-representation analysis of GO terms shows that Jiaxian Red has increased GO categories involved in cysteine-type endopeptidase inhibitor activity involved in apoptotic process (*TNFAIP8*, *DPEP1*, *SNCA*), regulation of microtubule polymerization (*SLAIN2*, *MAPRE1*, *SNCA*) (Table S9).

**Fig. 3:**
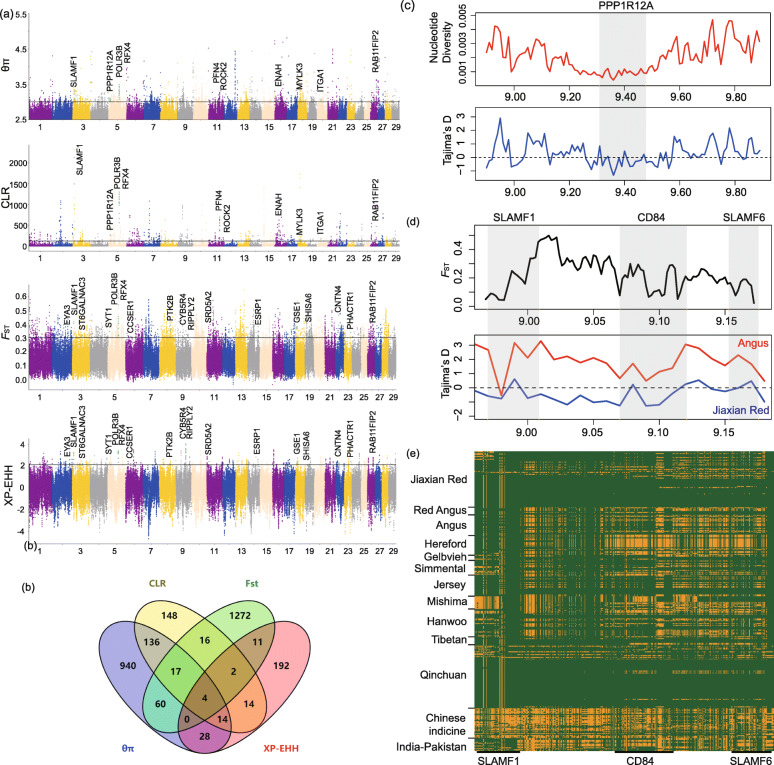
Analysis of the signatures of positive selection in the genome of Jiaxian Red. **a** Manhattan plot of selective sweeps in Jiaxian Red. **b** Venn diagram showing the genes overlap among θπ, CLR, *F*_ST_ and XP-EHH. **c** Nucleotide diversity and Tajima’s D at the *PPP1R12A* gene region. **d** *F*_ST_ and Tajima’s D plots of the *SLAMF1*, *CD84* and *SLAMF6* genomic regions. **e** SNPs with minor allele frequencies > 0.05 are used to construct haplotype patterns (Chr 3: 8.97–9.18 Mb). The major allele at each SNP position in Jiaxian Red is colored in yellow, the minor one in green

*F*_ST_ and XP-EHH test were also performed to detect the positive selection signatures between Jiaxian Red and the commercial breeds (Angus and Red Angus) (Fig. 3a). Based on the analysis, we obtained 1382 and 982 putatively advantageous positively selected genes from *F*_ST_ and XP-EHH methods, respectively (Tables S10 and S11); of these, 238 genes were detected in both methods and 17 genes were potentially selected candidate genes in Jiaxian Red (Table S12). Among these, strong signals of differentiation were obtained in the regions containing well known candidate genes related to meat quality traits (*CYB5R4*, *EYA3*, *PHACTR1*) and feed efficiency (*CCSER1*) [23–26]. It’s worth noted that four overlapped genes (*POLR3B*, *RAB11FIP2*, *RFX4* and *SLAMF1*) were detected among the 4 mentioned selection methods, indicating that these genes were strongly selected in Jiaxian red cattle. Among them, *RFX4* associated with fertility [27, 28], *SLAMF1* involved in the immune system response [29], *POLR3B* related to the autosomal-recessive hypomyelinating leukoencephalopathy [30], and *RAB11FIP2* plays a role in the secretory pathway [31].

## Discussion

The characterization of population structure and genetic diversity is essential for genetic assessment, understanding of environmental adaptation, as well as utilization and conservation of cattle breed genetic resources. We explored the population genetic structure of Jiaxian Red cattle in the context of the cattle breeds/populations with potential ancestors [2]. As shown in the ADMIXTURE analysis (Fig. 1a), the ancestral contributions of Jiaxian Red cattle came from East Asian taurine (~ 49%), Chinese indicine (~ 38%), European taurine (10%) and Indian indicine (~ 3%). Jiaxian Red is closest related to the Qinchuan breed, both of which are located in the central area of China, but Jiaxian Red shared more *Bos indicus* ancestry.

The nucleotide diversity of Jiaxian Red (mean θπ = 0.0029) was lower than that of Chinese indicine cattle (mean θπ = 0.0037), but approximately two times higher than that of European breeds (0.0010~0.0013). The highest genetic diversity observed in Chinese indicine cattle was in agreement with the results of Chen et al. (2018) [2], who reported the presence of Banteng (*B. javanicus*) introgression in Chinese indicine. The relative high level of genomic diversity found in Jiaxian Red is likely the results of hybridization with taurine and indicine, and may also reflect the weaker and shorter selection history. Jiaxian Red (mean θπ = 0.0029) and Qinchuan cattle (mean θπ = 0.0026) showed a similar level of nucleotide diversity (Fig. 2), which may be related to their similar genetic background. In addition, the patterns of LD decay in each breed was largely consistent with the results of nucleotide diversity. We also analyzed the ROH distribution pattern of Jiaxian Red by comparing with other cattle breeds. ROH are common in cattle autosomes, but the observed breed differences in the patterns of ROH length and burden suggested differences in breed origins and recent management [17]. Our results showed that Jiaxian Red exhibits larger amounts of short/medium ROH (0.5 to 2 Mb) and the lowest quantities of average ROH in comparison to the cattle breeds analyzed in this study, which is consistent with the reported ROH patterns of other taurine-zebu hybrid breeds [17].

In our analysis, Jiaxian Red and Qinchuan cattle showed a very close genetic relationship. In order to understand the genetic differences between the two breeds, we performed the GO enrichment analysis of genes harboring > 5 specific nsSNPs. Genes related to the immunity and “Sensory perception” were enriched in Jiaxian Red, which reflects the adaptability of Jiaxian Red cattle to environment. In addition, we also identified significant signatures of selective sweeps in Jiaxian Red breed. Jiaxian Red has been intensively bred for beef during over the past 30 years, leading to the genetic improvement in production traits, especially the characteristics of marbled meat. Marbling (intramuscular fat) is a valuable trait that impacts on meat quality and an important factor determining price of beef in the beef market. Jiaxian Red genome showed signs of selection in some genes of the “Regulation of actin cytoskeleton” pathway (*ITGA1*, *ENAH*, *MYLK3*, *ROCK2*, *PFN4*, *PPP1R12A*), which plays an important role in meat quality traits [20–22]. To better understand possible explanations for the selective pressures, we have explored the most likely biological functions of these genes. For example, protein phosphatase 1 regulatory subunit 12A (*PPP1R12A*), also known as *MYPT1*, is an important protein widely expressed in various cell types and plays a role in skeletal muscle insulin signaling [32]. *PPP1R12A* has been reported to be one of the highly expressed genes in intramuscular adipose tissue in the pig [33], and it has also been found under positive selection in Hanwoo (a famous beef cattle breed in Korea) [34], indicating that this gene may be related with meat marbling (intramuscular fat content) of Jiaxian Red (Fig. 3c). *ROCK2*, a gene that regulates cytokinesis, smooth muscle contraction and the formation of actin stress fibers, is involved in pathway associated with muscle/adipose tissue function in pigs with divergent phenotypes for fatness traits [35]. There are limited available publications to study this gene in cattle, but relative *ROCK1* gene is known for meat tenderness [36] and intramuscular fat (IMF) [35]. *ROCK1* gene has been considered one of the candidate genes related to IMF under selection in Ankole cattle [5].

When comparing the selection signatures of Jiaxian Red cattle with commercial breeds, two positively selected genes associated with muscle development (*PHACTR1* and *EYA3*) and one gene (*CYB5R4*) influencing the fatty acid metabolism have been identified in Jiaxian Red. *PHACTR1* is a member of the phosphatase and actin regulator family, which is involved in regulating the reorganization of the actin cytoskeleton. It has been reported in the literature that *PHACTR1* is a genetic susceptibility locus for fibromuscular dysplasia (FMD) [25]. *EYA* genes are associated with the proper development of muscles [37, 38]. *EYA3*-knockout mice exhibited the reduced movement [24]. *CYB5R4* is an electron donor for fatty acid desaturation by stearoyl-CoA desaturase (SCD) [39]. The C18 desaturation index (C18: 1/C18: 0) of *CYB5R4*-knockout mice was significantly reduced [40]. This gene has also been reported as one of the candidate genes for the QTL for oleic acid percentage in Japanese Black cattle [23], indicating that *CYB5R4* may be related with meat tenderness of Jiaxian Red. Feed efficiency is an important economic feature that affects beef production costs [41]. We detected positively selected gene *CCSER1* in Jiaxian Red, which was reported to be associated with the growth and feed efficiency in beef cattle [26].

Chinese native cattle are generally more resistant to disease than their commercial counterparts. Our selection analysis identified several genes involved in the immune system, in particular the *SLAMF1* gene that overlapped among the four selection methods. We found a region on BTA3: 8.97–9.18 Mbp containing three SLAMF (signaling lymphocytic activation molecule family) genes (*SLAMF1*, *CD84* and *SLAMF6*) that showed a strong signal of positive selection in Jiaxian Red. The positive selection signals around this region are further confirmed by significantly lower values of Tajima’s D and long haplotype patterns in Jiaxian Red (Fig. 3d and e). SLAMF receptors are involved in the regulation and interconnection of both innate and adaptive immune responses [29, 42]. The result suggested that this region may be one of the candidate regions of high disease resistance of Chinese native cattle, which may be useful as a genetic target for improving disease resistance in cattle breeding. But the haplotype of Jiaxian Red in this region is obviously not fixed, and similar haplotype pattern exists in Qinchuan cattle. This further suggested that Jiaxian red cattle did not experience the same intensive artificial selection as European cattle breeds. In addition, two genes (*RFX4*, *SRD5A2*) associated with fertility [27, 28] displayed signals of positive selection in Jiaxian Red cattle. These genes might be related to the strong reproductive performance of Jiaxian Red cattle.

Jiaxian Red breed is a valuable and widely used genetic resource in local area due to its higher beef productivity and better adaptability. As cattle genetic resources are being depleted and given the importance of this vital genetic resource, designing breeding programs that would help improve and conserve Chinese native cattle is crucial. With this regard, our results provide a basis for further research on the genomic characteristics of Jiaxian Red cattle in relation to economically important traits.

## Conclusions

This study provided a comprehensive overview of genomic variations in Jiaxian Red cattle by using WGS data. The characterization of population structure and genomic diversity will point out the direction for genetic assessment and development of reasonable breeding strategies of Jiaxian Red cattle. Moreover, we identified a series of candidate genes that may be important for the meat quality traits, growth and feed efficiency, immune response and fertility of this breed. These results provide a basis for further research on the genome characteristics of other important local beef cattle in the world.

## Methods

### Samples and sequencing

Thirty blood samples of Jiaxian Red cattle were collected from the core breeding farm of Jiaxian Red Cattle Breeding Center (Table S1). The animals were released after sampled. Genomic DNA was extracted by the standard phenol-chloroform method [43]. The paired-end libraries with the average insert size of 500 bp were constructed for each individual, with an average read length of 150 bp. Sequencing was performed using Illumina NovaSeq instruments at Novogene Bioinformatics Institute, Beijing, China.

To explore the ancestry proportions of Jiaxian Red and compare the genetic diversity with worldwide cattle breeds, we collected additional 131 samples according to the five “core” groups proposed by Chen et al. (2018) [2]. These samples include European cattle breeds (Hereford (*n*=10), Red Angus (*n*=5), Angus (n=10), Simmental (*n*=8), Limousin (n=1), Gelbvieh (*n*=3) and Jersey (*n*=11)), Korean native breed (Hanwoo, *n*=15), Japanese native breed (Mishima, *n*=7), Chinese native breeds (Tibetan (*n*=4), Qinchuan (*n*=34), Leiqiong (n=3), Guangfeng (n=4), Ji’an (n=4), Wannan (n=5)) and India-Pakistan cattle breeds ((Tharparkar (n=1), Sahiwal (n=1), Hariana (n=1), Nelore (n=1), Gir (*n*=2) and unknown (n=1)) (Table S2). A total of 161 animal samples were used in this study. All samples were divided into six groups (European taurine, Eurasian taurine, East Asian taurine, Indian indicine, Chinese indicine and crossbreed) based on the reports of Chen et al. (2018) [2].

### Reads mapping and SNP calling

The Burrows-Wheeler Aligner BWA-MEM (v0.7.13-r1126) [44] with default parameters was used to align the clean reads to the *Bos taurus* reference assembly ARS-UCD1.2. Picard tools (http://broadinstitute.github.io/picard) was used to filter potential duplicates reads (REMOVE_DUPLICATES = true). The Genome Analysis Toolkit (GATK, version 3.6–0-g89b7209) was performed to detect single nucleotide polymorphisms (SNPs). The raw SNPs were called using the “HaplotypeCaller”, “GenotypeGVCFs” and “SelectVariants” of GATK. After SNP calling, we used the “VariantFiltration” to discard sequencing and alignment artifacts from the SNPs with the parameters “QD < 2.0, FS > 60.0, MQ < 40.0, MQRankSum < -12.5, ReadPosRankSum < -8.0 and SOR > 3.0” and mean sequencing depth of variants (all individuals) “<1/3× and >3×”. Based on the annotation file (https://ftp.ncbi.nlm.nih.gov/genomes/all/GCF/002/263/795/GCF_002263795.1_ARS-UCD1.2/GCF_002263795.1_ARS-UCD1.2_genomic.gff) of the *Bos taurus* reference genome, a transcript FASTA file for database was built using the retrieve_seq_from_fasta.pl module of ANNOVAR, and then the functional annotation for each SNP was performed using the table_annovar.pl module of ANNOVAR [45]. We performed GO analysis for genes containing specific non-synonymous SNPs (nsSNPs) using the Database for Annotation, Visualization, and Integrated Discovery (DAVID).

### Population structure and phylogenetic analysis

We pruned the SNPs in high levels of pair-wise LD using PLINK [46] with the parameter (−-indep-pair-wise 50 5 0.2) to perform principal component analysis (PCA) and ADMIXTURE analysis. PCA was conducted using the smartPCA program in the EIGENSOFT v5.0 package [47]. Population structure analysis was carried out using ADMIXTURE v1.3 [48] with kinship (K) set from 2 to 8 (Table S13). The unrooted NJ tree was constructed with PLINK using the matrix of pairwise genetic distances and visualized with MEGA v5.0 [49] and FigTree v1.4.3 (http://tree.bio.ed.ac.uk/software/figtree/).

### Genetic diversity, linkage disequilibrium and ROH detection

We used VCFtools [50] to estimate nucleotide diversity of each breed in window sizes of 50 kb with 50 kb increment. Linkage disequilibrium (LD) decay with physical distance between SNPs was calculated and visualized by using PopLDdecay software [51] with default parameters. ROHs were identified using the --homozyg option implemented in the PLINK, which slides a window of 50 SNPs (−homozyg-window-snp 50) across the genome estimating homozygosity. The following settings were performed for ROH identification: (1) required minimum density (−homozyg-density 50); (2) number of heterozygotes allowed in a window (−homozyg-window-het 3); (3) the number of missing calls allowed in window (−homozyg-window-missing 5). The number and length of ROH for each breed were estimated and length of ROH was divided into four categories: 0.5–1 Mb, 1–2 Mb, 2-4 Mb, > 4 Mb.

### Selective sweep identification

Genome scans for selection in Jiaxian Red cattle were performed using the following strategy. First, we detect the selection signatures within Jiaxian Red cattle using two different statistics, the nucleotide diversity (θπ) and the composite likelihood ratio (CLR) [52]. The nucleotide diversity was estimated based on a sliding window approach with windows of 50 kb and a step of 20 kb using VCFtools [50]. The CLR test was calculated for sites in non-overlapping 50 kb windows by using SweepFinder2 [53]. Empirical *P* values were calculated for π and CLR windows, and the overlap of the top 1% windows of each method were considered as candidate signatures of selection.

Second, we performed comparisons between Jiaxian Red versus commercial cattle breeds (Angus and Red Angus) using fixation index (*F*_ST_) and cross-population extended haplotype homozygosity (XP-EHH). *F*_ST_ analysis was calculated in 50 kb windows with a 20 kb step using VCFtools [50]. XP-EHH statistics based on the extended haplotype was calculated for each population pair using selscan v1.1 [54]. For the XP-EHH selection scan, our test statistic was the average normalized XP-EHH score in each 50 kb region. An XP-EHH score is directional: a positive score suggests that selection is likely to have happened in Jiaxian Red cattle, whereas a negative score suggests the same about reference population. Significant genomic regions were identified by *P-*value *<* 0.01. Genomic regions identified by at least two methods were considered to be candidate regions of positive selection. Tajima’s D statistic was computed by using VCFtools for each candidate gene.

To gain a better understanding of the gene functions and signaling pathways of the identified candidate genes, online GO and KEGG pathway enrichment analyses were conducted using KOBAS 3.0 [55]. The GO and KEGG pathways were considered to be significantly enriched only when the corrected *P* value was less than 0.05.

## Supplementary Information

**Additional file 1: Figure S1.** Venn diagram showing overlapping and unique SNPs between the different cattle breeds or groups. The numbers in the circle components show specific SNPs for each breed or overlapping SNPs among breeds or groups. (a) The unique and shared SNPs between Jiaxian and “core” cattle groups, (b) The unique and shared SNPs between Jiaxian and Qinchuan cattle.

**Additional file 2: Figure S2.** Gene ontology (GO) terms enriched in 617 genes containing specific nsSNPs of Jiaxian Red cattle (compared to Qinchuan cattle). Advanced bubble chart shows enrichment of differentially expressed genes in signaling pathways. Size and color of the bubble represent amount of differentially expressed genes enriched in pathway and enrichment significance, respectively.

**Additional file 3: Figure S3.** Gene ontology (GO) terms enriched in 88 genes containing specific nsSNPs of Qinchuan cattle (compared to Jiaxian Red cattle). Advanced bubble chart shows enrichment of differentially expressed genes in signaling pathways. Size and color of the bubble represent amount of differentially expressed genes enriched in pathway and enrichment significance, respectively.

**Additional file 4: Table S1.** Summary of sequencing data. **Table S2.** List of additional cattle samples for analysis of genetic background in Jiaxian Red cattle. **Table S3.** Distribution of SNPs identified in cattle breeds within various genomic regions annotated by ANNOVAR. **Table S4.** GO enrichment results for the genes containing specific nsSNPs > 5 in Jiaxian Red cattle. **Table S5.** GO enrichment results for the genes containing specific nsSNPs > 5 in Qinchuan cattle. **Table S6.** A summary of genes from θπ in Jiaxian Red. **Table S7.** A summary of genes from CLR in Jiaxian Red. **Table S8.** KEGG pathway analysis of Jiaxian Red candidate genes overlapped by θπ and CLR methods. **Table S9.** GO enrichment of Jiaxian Red candidate genes overlapped by θπ and CLR methods. **Table S10.** A summary of genes from *F*_ST_. **Table S11.** A summary of genes from XP-CLR in Jiaxian Red cattle. **Table S12.** A summary of genes from *F*_ST_ and XP-EHH in Jiaxian Red cattle. **Table S13.** Cross-validation (CV) errors for ADMIXTURE ancestry models with K ranging from 2 to 8.

## Data Availability

Sequences are available from GenBank with the Bioproject accession numbers PRJNA634989.

## Abbreviations

SNP: Single nucleotide polymorphism
WGS: Whole-genome sequencing
θπ: Nucleotide diversity
CLR: Composite likelihood ratio
*F*_ST_: Fixation index
XP-EHH: Cross-population extended haplotype homozygosity
GO: Gene Ontology
KEGG: Kyoto Encyclopedia of Genes and Genomes
NJ: Neighbor-joining
PCA: Principle component analysis
ROH: Runs of homozygosity
LD: Linkage disequilibrium
nsSNPs: Nonsynonymous SNPs
