# Supplementary figures and images for: Assessing genomic diversity and signatures of selection in Jiaxian Red cattle using whole-genome sequencing data
Source: BMC Genomics. 2021 Jan 9;22:43. doi: 10.1186/s12864-020-07340-0 (PMC7796570; doi:10.1186/s12864-020-07340-0)

(a)

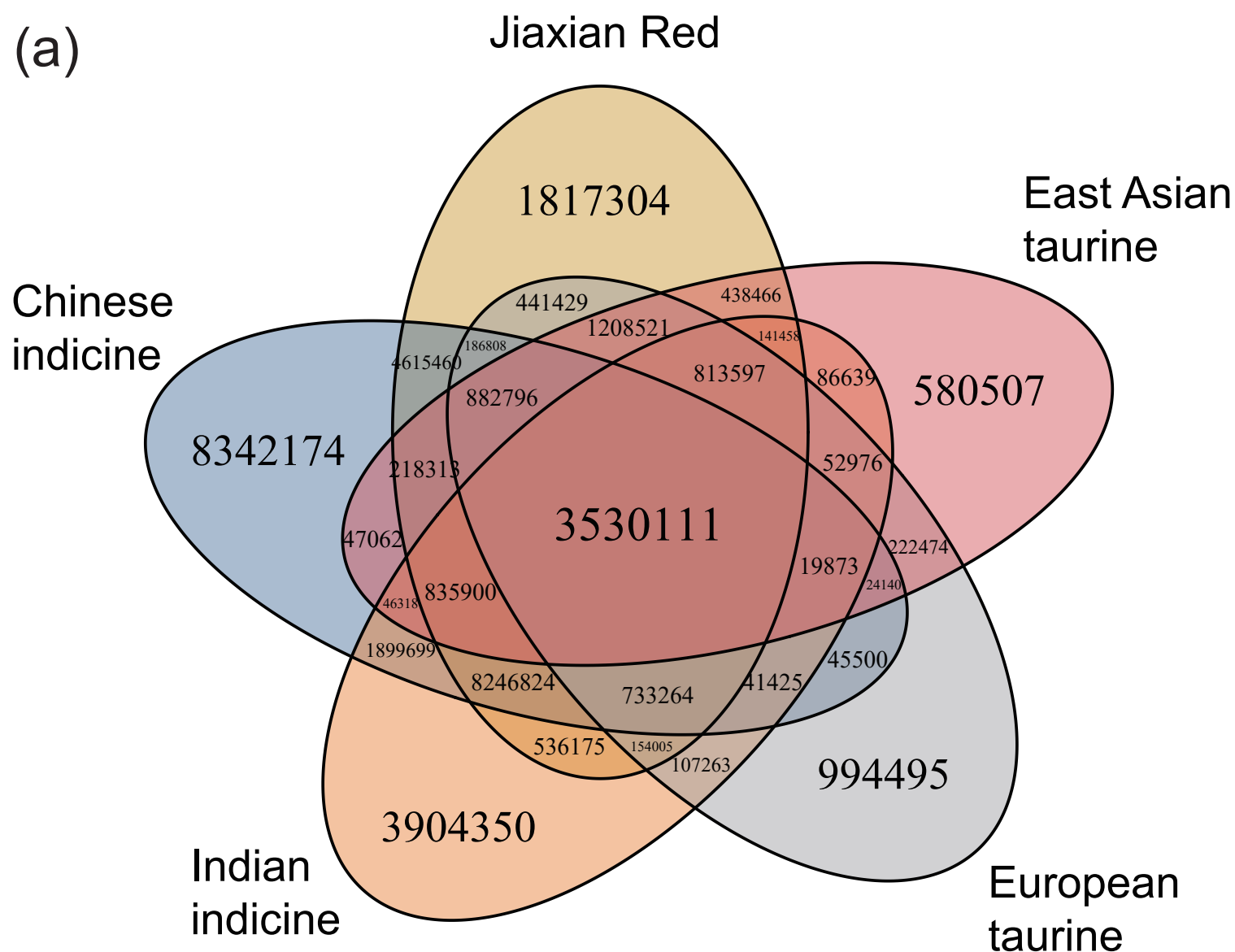

(b)

Jiaxian Red

Qinchuan

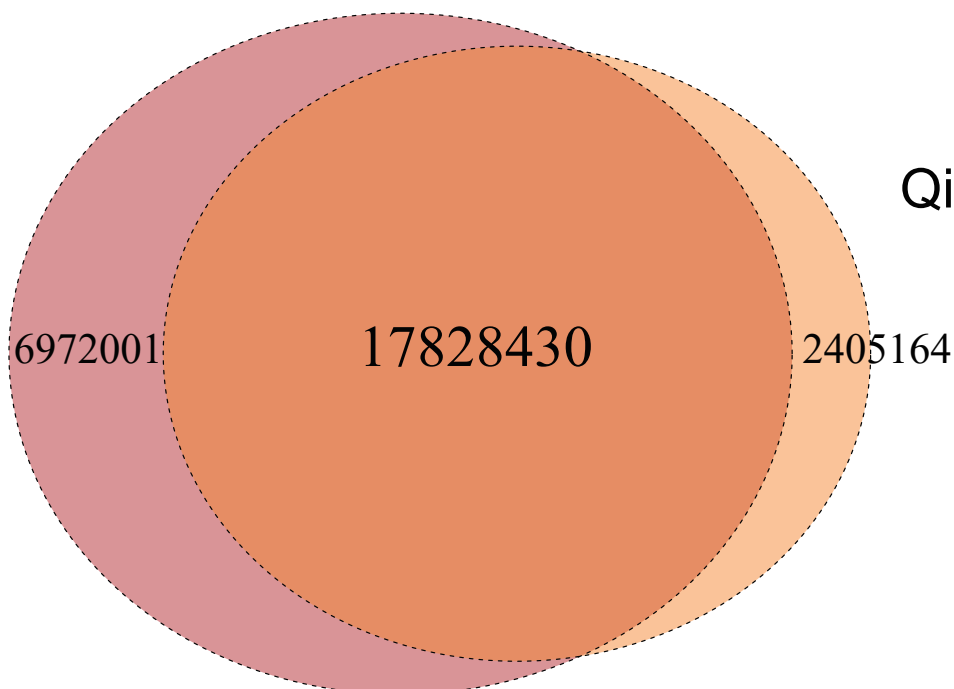

Supplement: Supplementary file 1 — Additional file 1: Figure S1. Venn diagram showing overlapping and unique SNPs between the different cattle breeds or groups. The numbers in the circle components show specific SNPs for each breed or overlapping SNPs among breeds or groups. (a) The unique and shared SNPs between Jiaxian and “core” cattle groups, (b) The unique and shared SNPs between Jiaxian and Qinchuan cattle. [file 12864_2020_7340_MOESM1_ESM.pdf]

GO term

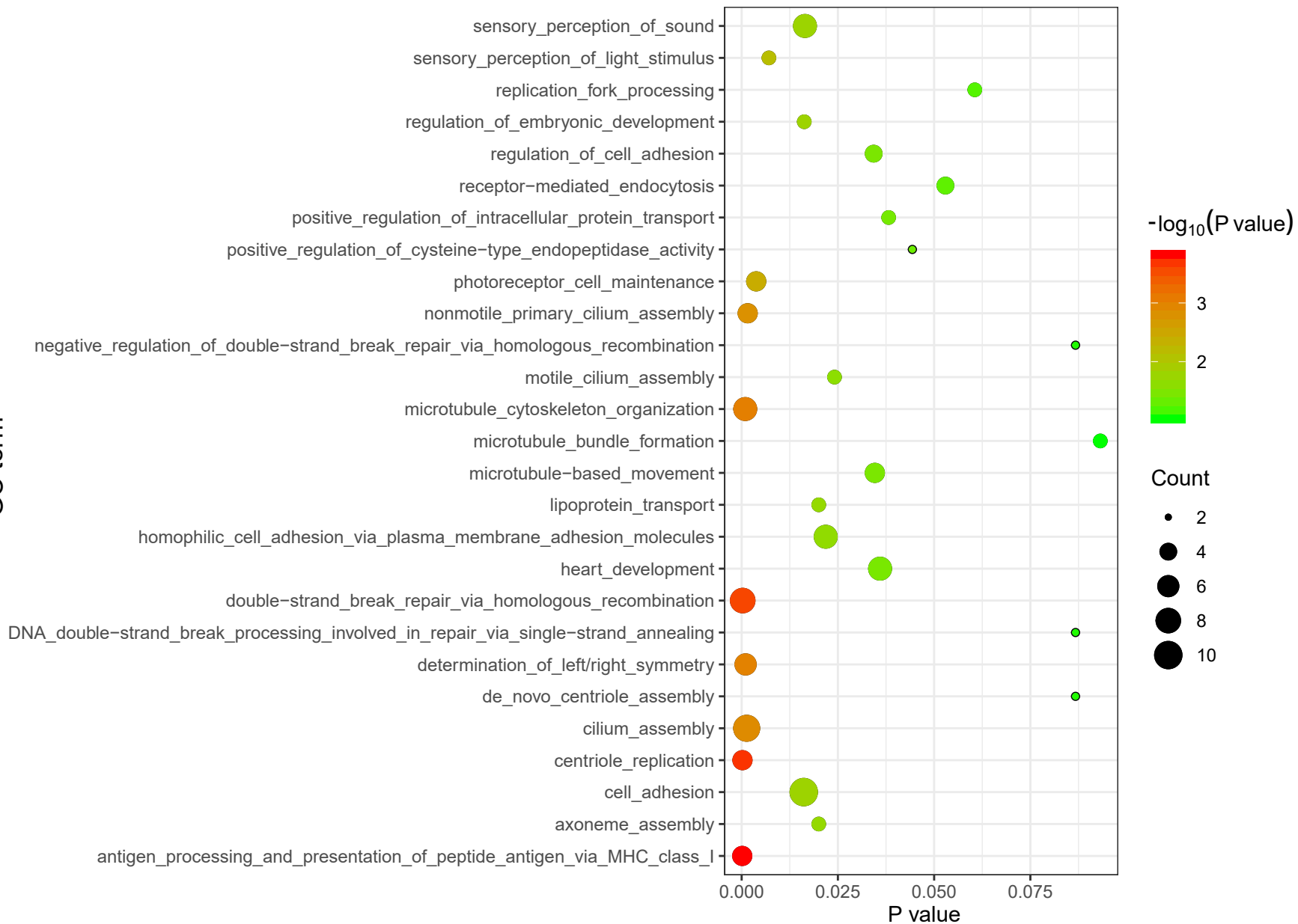

Supplement: Supplementary file 2 — Additional file 2: Figure S2. Gene ontology (GO) terms enriched in 617 genes containing specific nsSNPs of Jiaxian Red cattle (compared to Qinchuan cattle). Advanced bubble chart shows enrichment of differentially expressed genes in signaling pathways. Size and color of the bubble represent amount of differentially expressed genes enriched in pathway and enrichment significance, respectively. [file 12864_2020_7340_MOESM2_ESM.pdf]

GO term

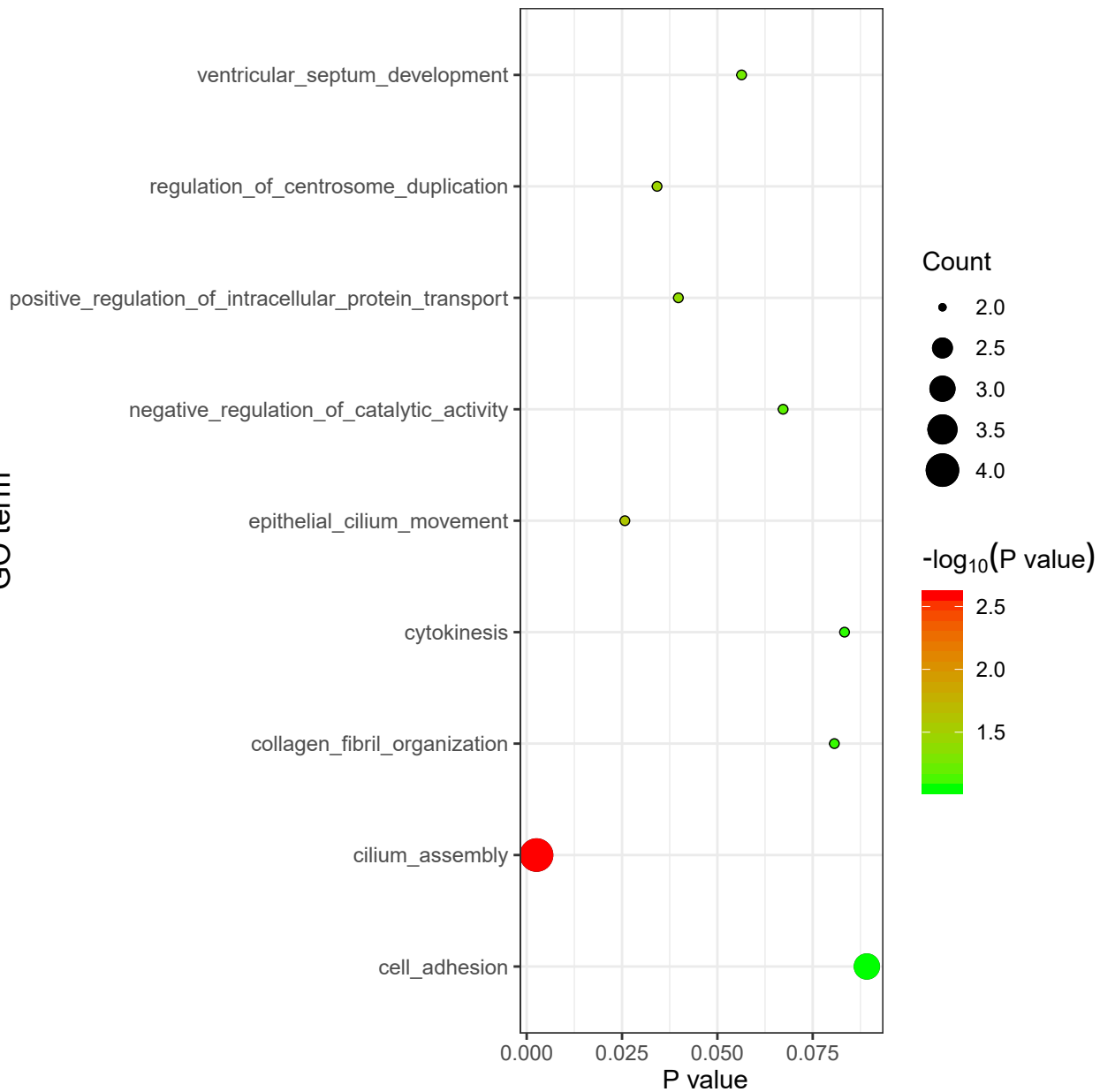

Supplement: Supplementary file 3 — Additional file 3: Figure S3. Gene ontology (GO) terms enriched in 88 genes containing specific nsSNPs of Qinchuan cattle (compared to Jiaxian Red cattle). Advanced bubble chart shows enrichment of differentially expressed genes in signaling pathways. Size and color of the bubble represent amount of differentially expressed genes enriched in pathway and enrichment significance, respectively. [file 12864_2020_7340_MOESM3_ESM.pdf]
